# Translating the learning sciences into practice: A primer for clinical and translational educators

**DOI:** 10.1017/cts.2021.840

**Published:** 2021-08-19

**Authors:** Marie K. Norman, Gaetano R. Lotrecchiano

**Affiliations:** 1 Innovative Design for Education and Assessment (IDEA) Lab, Institute for Clinical Research Education, University of Pittsburgh, Pittsburgh, PA, USA; 2 Department of Clinical Research and Leadership, Instructional Core for Advocacy, Research, and Excellence In Teaching and Learning (ICare), George Washington University School of Medicine and Health Sciences, Washington, DC, USA

**Keywords:** Learning science, education, translation, teaching, pedagogy

## Abstract

The learning sciences have yielded a wealth of insights about the mechanisms and conditions that promote learning, yet the findings from this body of research often do not make their way into educational practice. This fundamentally translational problem is one we believe that educators from translational fields, with their evidence-based orientation and familiarity with the challenges and importance of translation, are well-positioned to address. Here, we provide a primer on the learning sciences to guide educators in the Clinical and Translational Science Institutes and other organizations that train translational researchers. We (a) describe the unique teaching and learning environment in which this training occurs, and why it necessitates attention to learning research and its appropriate application, (b) explain what the learning sciences are, (c) distill the complex science of learning into core principles, (d) situate recent developments in the field within these principles, and (e) explain, in practical terms, how these principles can inform our teaching.

## Introduction

Almost a century of research on the brain and cognition has yielded a wealth of insights into how people learn – insights that can inform how we teach and train learners [1,2]. With a deeper understanding of the factors that affect learning, from the mechanics of memory to the conditions that spark and sustain motivation to the role of emotion in cognition, one would expect educators to be better positioned than ever before to teach effectively. Yet, it is disconcerting how inconsistently learning research makes its way into educational practice [3–10].

Disconcerting, perhaps, but is it surprising? As academics involved in translational research, we know all too well that the process of moving ideas from basic research in controlled conditions to application in the complex, messy real world is far from easy or automatic [11–13]. To do so effectively, bridges need to be built: key operational principles must be identified [11], complexity must be grappled with [12], technical language must be deciphered and made comprehensible to stakeholders [14], facilitators to implementation must be identified and barriers removed [15,16]. We also understand the critical importance and high stakes of translational pursuits and know that, without these bridges, important findings and insights from basic research languish in technical journals and are never used to improve practice or policy [17].

What’s more, because of our familiarity with the processes of translation and implementation, we may be especially well-suited to the work of bringing learning research into educational practice. We are evidence-based in our orientation, accustomed to working across disciplines to find effective approaches to complex problems, trained to find bridges between research and application, and firmly committed to educating and training the next generation of researchers. Moreover, because we are facing seismic shifts in the educational environment – a sudden move to remote and hybrid modalities, changing student populations, and an ever-broadening range of educational technologies – that demand constant innovation and adaptation [18–20], we understand the need to build new educational practices on a solid, evidence-based foundation.

Our goal in this article is to provide a primer on the learning sciences – new for some; a review for others – to guide us in this fundamentally translational process. We will (a) describe the teaching and learning environment in which translational researchers are trained, and explain why it necessitates an understanding of learning research, (b) explain what the learning sciences are and why they matter, (c) distill the complex science of learning into a set of basic principles, (d) situate recent developments in the field within these principles, and (e) explain, in practical terms, how these principles and insights can inform teaching and learning in our unique educational environment.

## Our Unique Educational Environment

Since their creation in 2006, the Clinical and Translational Science Institutes (CTSIs) have played a vital role in training and supporting the next generation of clinical and translational researchers [21–24]. Although surprisingly little has been written about learners in the CTSIs and in other organizations that train the translational workforce, we know that learners include graduate students, residents, fellows, faculty, research staff, and community collaborators [25]. They bring with them considerable prior education (bachelor’s degrees at minimum and often medical, master’s, and doctoral degrees) as well as deep expertise in their fields [26,27]. They tend to have concrete goals for their learning and seek the development of specific, practical skills. Educators also come from a wide variety of fields and departments, from surgery to social work to engineering. The same people who occupy the roles of teacher and student in one context may be colleagues and collaborators in another context, creating a somewhat flattened hierarchy atypical of academic medicine [28,29]. This promotes a high degree of collegiality among instructors and learners.

Translational science is taught in a range of contexts, including credit-bearing courses in master’s, PhD, and certificate programs; training programs for early career investigators; professional development workshops and seminars in areas such as mentorship [30,31], leadership [32–34], equity and inclusion [35,36], teamwork and team science [37–39]; and in the informal space of mentor–mentee relationships and interdisciplinary collaborations [40]. Both formal and informal curricula in the CTSIs tend to be practical rather than theoretical, focused on skill-building in discrete competency areas (e.g., statistical knowledge, grantsmanship, qualitative research skills [41,42]). Since the COVID-19 pandemic, we – along with the rest of higher education – have seen a shift in learning modalities toward online and hybrid programming that may become more permanent [18,43].

These unique elements of the teaching and learning environment, in particular the focus on teaching adult learners in diverse and generally interdisciplinary contexts, should be foremost in our minds as we consider how to apply the rich science of learning to our educational pursuits.

## What Are the Learning Sciences and Why Do They Matter?

For such a widely used term, “learning” has proven remarkably difficult to pin down [44,45]. Most definitions describe a process of change, prompted by experience, that increases knowledge [46]. It is not a change that happens to learners passively but rather something that learners must *make* happen by reflecting on the experience and forming and testing mental models [47,48]. Learning is understood to be an interior process that cannot be measured directly but must be inferred through behavior [49]. Performance, in other words, serves as a proxy for learning. Many researchers situate the locus of learning within individuals; however, others locate learning in social interactions [1,48,50–52], a formulation that has been extended to describe learning at the level of teams and organizations [53,54].

The term “learning sciences” emerged in the 1990s to describe an interdisciplinary field of research that seeks to understand the mechanisms by which learning occurs in real-world situations and to identify and encourage practices that facilitate learning [55,56]. The learning sciences are inherently interdisciplinary, drawing on a diverse array of fields including cognitive and developmental psychology, neuroscience, computer science, sociology, and anthropology [57].

Among other things, the learning sciences have challenged long-standing myths about teaching and learning [58–61]. Among these myths is the belief that subject matter expertise is sufficient to make one an effective teacher [62], that increasing content increases learning [63,64], that lecturing by itself is an effective teaching strategy [4,65], and that it is important to diagnose and teach to specific learning styles [59,66,67]. None of these beliefs is supported by evidence. Teaching requires knowledge and skills entirely distinct from subject matter expertise. Less content, accompanied by opportunities for active engagement, contributes to deeper learning and longer retention [4]. Similarly, lectures yield poor learning results relative to active learning, and should be used advisedly [4,68]. Moreover, although many educators tout the importance of adjusting their teaching strategies to students’ individual learning styles, there is little in the research literature to support that approach. Indeed, “learning styles” are generally little more than context-dependent preferences and not stable states; thus, researchers agree that instructors are better off adjusting their teaching strategies to the content rather than to students’ professed learning styles [59,69–72].

In addition to expanding research on learning and debunking myths, the learning sciences have sought to distill existing research (often highly technical in its original form) into core principles and practical strategies to guide teaching practice. These distillations have yielded principles of adult learning [73], principles to promote deeper learning and knowledge retention [74–76], multimedia design principles [77], principles of social learning [78], and theories of applied intelligence [79], among others. Each framework organizes the complex literature in somewhat different ways, with different foci and intended audiences, and all are valuable. For the purposes of this article, we have loosely adapted the framework set out in Ambrose et a l[57]. This framework, which synthesizes half a century of literature on learning, identifies a basic set of principles to help educators understand how learning works, as well as how to use that understanding to teach more effectively. The principles are not specific to any discipline or student level, and thus apply across learning contexts and modalities. Moreover, they are sufficiently broad to encompass new discoveries and formulations, such as work in the areas of cognitive load and social presence, which we have also included.

For simplicity, we have organized these principles into three categories: *acquisition and integration of knowledge, social and emotional components of learning*, and *elements of skill-building*. In the following sections, we describe the research that informs each area and explain how it relates to the specific learning environments in which translational researchers are educated.

## Acquisition and Integration of Knowledge

Four areas of the learning sciences shed light on how knowledge is acquired and integrated. They concern the role of prior knowledge, knowledge organizations, cognitive load, and metacognition.

### Prior Knowledge

All learning builds on prior knowledge [80–82]. Indeed, learning *only* occurs when learners connect what they are learning to what they already know or have experienced. In the case of adult learners, who bring significant academic, professional, and life experience into new learning situations, there is a strong knowledge foundation on which to build and one that educators should not neglect. However, gaps and deficits in prior knowledge can also impede learners’ ability to integrate new knowledge and may be particularly important to recognize and address in interdisciplinary learning environments, where students and trainees come from different academic and professional backgrounds and do not all possess the same baseline knowledge. The interdisciplinarity of institutions and departments focused on translational education may also create other learning challenges, including the inappropriate application of prior knowledge. Specifically, learners may apply knowledge gained in one context (e.g., prior degree programs) in contexts where it is not relevant or applicable [57]. (One example, for instance, is importing concepts of bias and generalizability from quantitative fields into qualitative research, which operates on very different terms.) Both knowledge gaps and misapplied prior knowledge are issues that educators should be aware of and look to remediate.

- *Advice for educators: Help learners connect what they are learning to what they already know and have experienced, but also pay close attention to – and address – what they do not know, apply in the wrong context, and believe in error*.

### Organization of Knowledge

Learning involves not only *what* learners know but how they organize what they know. The ways that knowledge is organized determines how easily it can be retrieved and how effectively it can be used [57,83]. However, the organizational frameworks of experts and novices differ markedly [84,85]. Expert knowledge is richly connected [85], making it possible for experts (including teachers) to readily see how ideas are linked. Moreover, experts organize what they know around the deep structures and underlying principles of problems and cases, rather than superficial similarities [83]. Experts also possess multiple organizational frameworks, which allow them to sort information in different ways for different purposes and facilitates the transfer of that knowledge to new situations [57]. Expert/novice differences are important to recognize in the context of teaching and learning. As experts in their fields, educators – even at the graduate level – cannot assume their learners naturally possess these organizational structures. Rather, part of the task of educators is to help learners develop similarly meaningful and flexible knowledge organizations [86,87].

- *Advice for educators: In addition to imparting information, provide organizational frameworks and schemas to help learners organize their growing knowledge in meaningful and practical ways. Also, ask questions that require learners to make and articulate connections, thus growing their neural networks [88]*.

### Cognitive Load

Recognition of the limitations of working memory has been one of the most important discoveries to come out of the learning sciences [89]. Working memory is the cognitive system responsible for manipulating, encoding, and organizing new information before it is ultimately moved into long-term memory. While long-term memory is capacious, with almost limitless space (think: the Library of Congress), the cognitive resources available for processing information in working memory are highly limited (think: your physical desktop) and must be husbanded carefully. Cognitive load theory focuses on ways to make optimal use of working memory for learning [90,91]. Scholars in this area have differentiated between intrinsic, germane, and extraneous cognitive load. *Intrinsic cognitive load* refers to the cognitive resources required by a task itself (e.g., reading a journal article). *Germane cognitive load* refers to the cognitive resources required to generate meaningful connections or develop a schema (e.g., connecting the content of one journal article to others). *Extraneous cognitive load* refers to cognitive resources eaten up by incidental or unnecessary factors (e.g., the confusing directions of an instructor) [92,93]. Learning scientists agree that instructors should minimize extraneous cognitive load while maximizing germane cognitive load [92], in other words, to make sure the difficulty in a task advances learning without draining cognitive resources unnecessarily. Cognitive load theory is particularly applicable in the context of online learning, where poorly organized platforms and unfamiliar technologies can add extraneous cognitive load, potentially eroding motivation and impeding learning [94–96].

- *Advice for educators: Increase germane cognitive load by assigning tasks and asking questions that compel learners to think harder about the material you are teaching. At the same time, decrease the extraneous cognitive load by making written directions clear and succinct and employing good visual design*.

### Metacognition

Another critical facet of knowledge acquisition is metacognition or the process by which learners understand, monitor, and refine their own cognitive processes [97–99]. Ambrose et al represent metacognition as a set of five abilities [57]: first, the ability to realistically and accurately *assess the requirements of a task* (e.g., the time, resources, and skills required); second, the ability to *evaluate one’s own skills and competencies* relative to the task requirements; third, the ability to *plan* appropriately; fourth, the ability to *monitor* and assess performance as one acts; and fifth, the ability to *reflect* back on one’s performance after the fact and make adjustments for the future. While one would think that accomplished graduate-level learners typical of the CTSIs and other translational educational contexts have already developed strong metacognitive skills, research indicates that, in fact, adult learners often fail to monitor their own thinking and fall back into familiar patterns and biases that limit their intellectual growth [100]. Research also shows that metacognitive skills can be strengthened considerably and with very positive outcomes for learning if instructors provide structured opportunities for self-evaluation, planning, and reflection on past performance [101,102].

- *Advice for educators: Allocate ample time for learners to reflect on their strengths and weaknesses in relation to complex tasks, to assess the demands of those tasks, and to plan their strategy. Allow time at the mid-point of projects for learners to stop, monitor progress, and adjust their approach, and leave time at the end of such tasks for learners to reflect on their performance and plan*.

## Social and Emotional Components of Learning

Learning is an intensely communal activity that cannot be divorced from the social and interactive contexts in which it occurs [48,51,103]. Indeed, there is increasing recognition that learning is heavily influenced by social and emotional – and not simply cognitive – factors [104], a fact that is even more apparent since the advent of online education [105,106], where social connection and community can become attenuated, with detrimental impacts on learning. There is far more to say about the social elements of learning than space here allows. However, four important principles concern the factors that influence *motivation*, the importance of learners’ *developmental stage*, the ways in which *climate* affects learning, and the role of *presence*, particularly online.

### Motivation

Motivation drives the behaviors that result in learning and is thus a critical ingredient in all learning contexts. There are two high-level factors that, taken together, increase learner motivation: *value* and *expectancy* [57,107]. Value stems from learners’ perceptions that the material they are learning and the tasks they are engaged in are relevant, meaningful, and of practical value. According to the tenets of self-determination theory, three elements increase perceived value: *competence* (awareness of increasing mastery), *relatedness* (connection and accountability to other people), and *autonomy* (a sense of agency and control) [108]. Daniel Pink adds to that a sense of *purpose* [109].

The other factor in motivation is expectancy. Expectancy concerns learners’ beliefs that success is possible: that their efforts are connected to desired goals [110], that they are personally capable of achieving those goals [111], and that the environment will support and not thwart their efforts [57]. Learners who believe that a task is unreasonably difficult, that they are personally incapable, or that they do not have adequate support will lose motivation. Both value and expectancy must be present for motivation to be high. If learners value an outcome but do not feel capable of achieving it (high value, low expectancy) they will lose motivation. By the same token, if learners feel capable of achieving a goal but do not value it (low value, high expectancy), motivation will suffer. Notice that both value and expectancy are issues of perception, not objective reality: learners must *believe* that what they are learning has value and that successful learning is possible. While graduate-level learners often possess a fair degree of intrinsic motivation, instructors should not assume that their motivation will be high for all tasks and activities or that motivation cannot be eroded even when initially high. Considering ways to increase value and expectancy is thus a wise course of action for all educators.

- *Advice for educators: Seek to increase learners’ motivation by highlighting the practical value of what they are learning and reducing factors that erode expectations of success, without compromising high standards. Provide opportunities for learners to exercise autonomy, demonstrate increasing competence, and connect with one another*.

### Developmental Stage

While the factors that affect learning (e.g., prior knowledge, motivation, metacognition) are the same for students at all life stages, learners themselves differ, as do their learning needs [57]. Various stage models have been offered to help educators understand learners at different phases of life. These include Perry’s model of intellectual development, which describes four stages in learners’ ability to tolerate ambiguity and countenance different perspectives on complex issues yet, ultimately, commit to action [112]. Perry’s model has been refined and extended by Baxter-Magolda, who has explored the issue of “self-authorship” across cognitive, interpersonal, and intrapersonal domains of development [113]. Stage models also include theories of racial identity development [114–117]. While many such models focus on the developmental tasks of traditional college-aged learners [118,119], the paradigm with perhaps the most relevance to the educational context of the CTSIs is Knowles’ theory of *andragogy* [120–122]: teaching adult learners. Theories of adult learning vary but the primary components are these: Adult learners want to know how the material they are learning serves concrete personal or professional goals [123,124]. They learn best by doing, i.e., through practice and participation, preferably through problem-solving [125]. They bring experiences to the learning encounter that can facilitate learning but also at times cause mental rigidity [1,120]. Finally, adult learners do best in informal environments, in which they have a degree of self-direction and control, and where the relationship between instructor and learner is more collaborative than directive [126]. It should be noted that developmental theories, many of which took individual psychology as their starting point and neglected structural issues of power and inequity, have been reexamined in recent years through the lens of critical theories about race, ethnicity, gender, and disability [119,127,128].

- *Advice for educators: Assign tasks with obvious practical relevance to learners’ professional and/or personal lives, focus on allowing students to learn by doing, allow ample opportunities for learners to bring their experiences to bear in discussion, and approach the learning situation in a collegial and collaborative manner*.

### Climate

Equity and inclusion are and should be an increasing focus within higher education [5,66]. A critical issue for educators to consider is whether the learning climate they foster in courses and training seminars is genuinely inclusive, welcoming, and supportive of diverse learners [129,130]. We know that when the climate of a classroom or training is overtly or subtly marginalizing toward learners, whether on the basis of race, gender, age, sexual orientation, disability, or any other factor, it exacts a high toll on learning, performance, motivation, and persistence [131–135]. Powerful messages about inclusion and exclusion can be conveyed to learners simply by the choice of authors and topics to include (or not include) on a course reading list [57]. Assumptions and biases about ethnic and racial groups can be embedded in case studies [136]. Choices in instructional materials (e.g., the use of videos without subtitles or podcasts without transcripts) can marginalize and disadvantage students with visual or auditory disabilities [137]. Microaggressions can be prevalent in classrooms and detrimental to students’ learning and persistence in the field [138–140]. A fascinating body of research on stereotype threat demonstrates that, when stereotypes are triggered even in the subtlest ways, members of stereotyped groups can experience a disruptive cognitive state that undermines learning and performance [141,142].

Fortunately, there is much that instructors can do to create inclusive learning environments, including employing simple strategies to reduce stereotype threat, such as communicating high expectations for all learners [143–145]. Other factors that create a positive learning climate are the demonstration of “instructor immediacy” – verbal and nonverbal instructor behaviors that convey approachability to students [146–149]. The communication of immediacy is particularly important online, where learners can easily feel isolated [150]. Universal Design for Learning (UDL), a set of guidelines that grew out of disability research, seeks to make learning accessible to all through the design of flexible learning environments in which learners have a range of choices in how they engage with instructional materials and demonstrate learning [151,152]. While the impact of UDL has yet to be empirically assessed, it is grounded in well-established learning research and early studies look promising [153].

- *Advice for educators: Work to create a learning environment that is intellectually challenging yet welcoming to every learner. Use content that reflects diverse voices and conveys approachability. Design for accessibility and inclusion*.

### Presence

Online learning has distinct benefits when it comes to convenience, access, and self-pacing; however, it also has challenges, principally the attenuation of social connection that comes when people are not physically “present” with one another. Scholarship coming out of the Community of Inquiry framework [154,155] has emphasized the importance of creating three types of “presence” in online courses: *social presence*: the ability of learners to project their identities and connect with one another effectively through technologically mediated means [156,157]; *cognitive presence*: the ability of learners to connect deeply to course content [158,159]; and *teaching presence*: the instructor’s ability to reach across the distance, seem real and genuine, and connect meaningfully with learners [150]. This body of research points to the fact that social connection and community building cannot be taken for granted but must be developed deliberately and cultivated carefully online [160,161]. As the CTSIs expand their online programming, this research is critically important to consider. However, the Community of Inquiry framework is equally applicable to face-to-face and hybrid educational environments and speaks to the powerful social and emotional components of learning.

- *Advice for educators: In all courses, but especially online, be deliberate about projecting your own personality and presence while working to build community and encourage meaningful interaction among learners*.

## Elements of Skill-Building

Considerable scholarship in the learning sciences has attended to the processes and stages by which learners acquire skills, gain fluency and automaticity using those skills, and develop expertise within a particular domain [162]. Much of the research in this area explores differences in the ways experts and novices organize, access, and use information [84,163] and is informed by research on artificial intelligence and machine learning. Two relevant principles relate to the development of *mastery* and the role of *practice and feedback* in that process.

### Mastery

To develop expertise in a given domain (say, clinical research), learners must master complex skills. According to Ambrose et al., this requires first that they acquire the component skills that make up the complex skill (consider, for example, how many sub-skills are required to perform a task like writing a grant proposal!). In addition to acquiring these sub-skills, learners must *integrate* them successfully, developing speed and fluency at executing these skills in combination. Finally, they must understand when and where to *apply* what they have learned [1,57]. This final element of mastery is also known as transfer and is, arguably, the central point of learning [164]. When should you employ a particular research design? When are specific statistical methods appropriate? Skill gaps at any of these levels can inhibit the development of mastery and interfere with performance. Ironically, one factor that complicates learning for relative novices is the expertise of their teachers, whether in formal or informal learning contexts. Because experts have gained mastery to the point of unconscious fluency [165], they tend not to see all the steps and component skills involved in learning complex tasks, and thus often do not scaffold tasks appropriately for learners. Researchers call this “expert blind spot” [166,167]. It is a hazard that educators in translational science programs should watch for, because their own expertise can sometimes blind them to the learning needs of students.

- *Advice for educators: Recognize that mastery takes time to develop and allocate sufficient time for students to learn skills in isolation, practice them in combination, and use them in diverse contexts to develop transfer. Also, watch out for your own expert blind spot when teaching others!*

### Practice

Practice and feedback are both essential for developing competence in any domain [57]. Practice without feedback is not only demotivating; it also reinforces mistakes [168–170]. Feedback without practice, on the other hand, is pointless: without opportunities to address mistakes, learners do not improve. They also lose motivation [171]. However, not all practice and feedback are useful. Ideally, the practice should be focused on specific performance goals [172,173]. The task, moreover, should be appropriately challenging: too easy and the learner is not pushed to improve; too difficult and both performance and motivation suffer [57,174,175]. Finally, the practice should involve sufficient time on task [176]. A fascinating area of research has focused on what Bjork and Bjork have called “desirable difficulties” [177–179]. As it turns out, when learners struggle to learn something, they encode the information more deeply and remember it longer. Thus, there is an optimal level of difficulty (challenging but not discouraging) that facilitates learning. A related area of research is on “retrieval practice,” also called the testing effect [177,178,180,181]. This scholarship has found that the act of retrieving information from long-term memory, whether through testing or simply by being asked questions, helps to create stronger mental paths back to that information and ultimately leads to deeper learning [60]. In addition, spacing practice sessions farther apart (the “spacing effect”) aids learning by compelling learners to engage in more effortful retrieval [182]. The research on retrieval practice and spacing has had particular resonance in medical education, where learners are expected to integrate and remember vast amounts of information [183].

- *Advice for educators: Make sure learners have ample and repeated opportunities to practice key skills, ensuring that tasks are sufficiently difficult to be effortful but not so difficult as to be discouraging. Give learners retrieval practice by asking frequent questions or giving low-stakes assessments*.

### Feedback

Feedback, or information provided to learners to help them improve their understanding or performance, is one of the most powerful factors affecting learning [62,184]. Feedback helps learners identify gaps between current and desired knowledge and skills while helping to identify specific actions they can take to close the gaps. Feedback helps learners develop stronger skills at self-evaluation [185] and also plays a key role in motivation [186]. Research shows that feedback is most effective when it focuses on specific areas for improvement [187], is prioritized so as to differentiate high-importance items from low-importance items [188], and is delivered soon after performance [184]. The collegial, mentorship-focused nature of education in the CTSIs makes feedback a particularly important tool for helping learners improve.

- *Advice for educators: Provide learners with feedback that identifies specific, actionable, and prioritized areas for improvement, make sure the feedback is delivered in a timely fashion, and ensure there are immediate opportunities for learners to incorporate your feedback into practice*.

## Recommendations

While the summary of research and advice for educators provided in the earlier sections may seem somewhat daunting, the lessons are actually simple and intuitive. Taken together, they suggest the types of strategies for teaching and training outlined in Table 1.

**Table 1.** Strategies educators can use to incorporate research-based learning principles

*How can educators help learners acquire and integrate knowledge more effectively?*
Prior knowledge: Do a short prior knowledge assessment at the beginning of courses or trainings to ensure you are starting at the right place and with appropriate pacing. Create opportunities for learners to bring their knowledge and experiences (personal and cultural as well as professional) to bear on what they are learning. Watch for knowledge gaps in key areas that may impede new learning, and remediate them. Explicitly address faulty assumptions learners might make on the basis of previous academic training or professional experiences.
Organization of knowledge: Pay attention to how you, as an expert in your field, organize information, and make these implicit organizational schemas explicit to your learners. Provide tables, templates, and other organizational scaffolding to help learners structure information in ways that facilitate appropriate application.
Cognitive load: Increase germane cognitive load by pressing learners to draw connections and elaborate meanings. Decrease extraneous cognitive load by making directions clear and organizing information for easy navigation.
Metacognition: Provide structured opportunities (as in-class activities or assignments) for learners to evaluate their own skills and abilities and identify areas for improvement. Allow sufficient time for project planning and encourage learners to take time mid-project to assess and modify strategies if necessary. Allocate time at the end of projects and major assignments for learners to reflect on their experience and identify what they would do differently in the future.
*How can educators better address the social and emotional components of learning?*
Motivation: Assign tasks with immediate practical relevance. Ask learners to identify the value of these tasks relative to their professional and personal goals. Make sure that instruction and assessments are well-aligned, that requirements are reasonable and fair, and that learners have ample time and support for challenging tasks to increase expectancies. Look for opportunities to give learners choices in assignments and responsibility for their work (autonomy). Highlight their growing mastery (competence) and encourage them to work collaboratively to build a connection with peers (relatedness).
Developmental stage: Give adult learners ample opportunities to share their expertise and discuss their experiences. Assign practical, hands-on tasks that align with real-world work products (e.g., a study design, manuscript abstract, research poster). Encourage peer-to-peer mentoring and peer review.
Climate: Communicate high standards and confidence that all learners can meet them. Choose materials (readings, case studies, images) that represent diverse experiences and avoid stereotypes. Seek ways for learners to bring their cultures and backgrounds into the classroom. Use Universal Design for Learning (UDL) principles when designing instruction to create greater inclusion.
Presence: Create opportunities for synchronous and asynchronous student-to-student interaction in online courses by using breakout rooms, discussion forums, group projects, social media, etc. Use methods in online courses (e.g., informal video updates, weekly email check-ins, online office hours) that make you present and accessible to learners.
*How can educators help learners build skills and gain mastery?*
Mastery: Deconstruct complex skills into their component parts, and make sure learners have adequate practice learning key sub-skills in isolation before asking them to use those skills in combination. Recognize your own expert blind spot, and work against it by making implicit knowledge (the steps and practices you intuitively engage in) explicit to learners.
Practice: Make sure that the tasks you assign are appropriately challenging: difficult enough to be cognitively demanding, but not so difficult as to erode motivation. Give learners multiple opportunities to practice key skills. Employ retrieval practice by frequently asking learners to recall and apply information learned on prior occasions. Use cumulative tests (which compel learners to engage in both retrieval practice and spacing) but give learners ample opportunities to use their knowledge before testing them.
Feedback: Move beyond compliments and criticism to provide specific, actionable information learners can use to improve performance. Provide feedback in a timely manner, when the performance it is based on is still fresh in learners’ minds. Leverage peer review to provide learners with feedback from more sources.

## Conclusion

Teaching and learning are ubiquitous in the CTSIs and other institutions focused on training the translational workforce. Thus, there is much for educators in the CTSIs to gain by cultivating a deep understanding of the mechanisms of learning and the attributes of high-quality teaching. In this article, we have made a case for bringing the learning sciences more systematically into our educational practices. We have argued, moreover, that educators in the field of translational science may be particularly well-equipped to translate the rich, varied, and interdisciplinary research on learning into practice because of their appreciation for the importance and complexity of translational pursuits, and their commitment both to evidence-based practices and to educational excellence.

We have offered this distillation of key principles from the learning sciences and contextualized it within our unique educational environment in the hope that this framework can provide helpful guidance and a shared vocabulary for educators at our institutions, regardless of the specific contexts in which they teach. We believe that, armed with these principles, educators will be better able to discern why effective practices are effective, identify and address teaching problems, adapt strategies successfully to new teaching contexts and modalities, and innovate from a solid foundation of understanding.
